# PAAR proteins act as the ‘sorting hat’ of the type VI secretion system

**DOI:** 10.1099/mic.0.000842

**Published:** 2019-08-05

**Authors:** Thomas E. Wood, Sophie A. Howard, Sarah Wettstadt, Alain Filloux

**Affiliations:** ^1^​ MRC Centre for Molecular Bacteriology and Infection, Department of Life Sciences, Imperial College, London, London, SW7 2AZ, UK; ^†^​ Present address: Department of Medicine, Division of Infectious Diseases, Massachusetts General Hospital, Cambridge, MA, USA; ^‡^​ Present address: Department of Microbiology, Blavatnik Institute, Harvard Medical School, Boston, MA, USA; ^§^​ Present address: Department of Environmental Protection, Estación Experimental de Zaidín – Consejo Superior de Investigaciones Científicas, Granada, Spain

**Keywords:** type VI secretion system, VgrG, PAAR, effector, immunity

## Abstract

Bacteria exist in polymicrobial environments and compete to prevail in a niche. The type VI secretion system (T6SS) is a nanomachine employed by Gram-negative bacteria to deliver effector proteins into target cells. Consequently, T6SS-positive bacteria produce a wealth of antibacterial effector proteins to promote their survival among a prokaryotic community. These toxins are loaded onto the VgrG–PAAR spike and Hcp tube of the T6SS apparatus and recent work has started to document the specificity of effectors for certain spike components. *Pseudomonas aeruginosa* encodes several PAAR proteins, whose roles have been poorly investigated. Here we describe a phospholipase family antibacterial effector immunity pair from *Pseudomonas aeruginosa* and demonstrate that a specific PAAR protein is necessary for the delivery of the effector and its cognate VgrG. Furthermore, the PAAR protein appears to restrict the delivery of other phospholipase effectors that utilise distinct VgrG proteins. We provide further evidence for competition for PAAR protein recruitment to the T6SS apparatus, which determines the identities of the delivered effectors.

## Introduction

Bacteria must influence their surroundings and compete with other micro-organisms for nutrients and space. The deployment of protein secretion systems such as the type VI secretion system (T6SS) facilitates the elimination of competitors and enables bacteria to establish a foothold within a niche. The T6SS is a prevalent Gram-negative bacterial virulence factor and antibacterial apparatus, delivering effector proteins directly into target cells or into the local environment [1–3]. Many effector proteins exhibit antibacterial activities, such as nucleases, phospholipases and peptidoglycan hydrolases, to induce stasis or lysis of the target bacterium [4]. The T6SS is a contractile apparatus that propels a lance-like structure decorated with effectors into neighbouring bacteria in a contact-dependent manner. The lance is composed of a tube of stacked haemolysin-coregulated protein (Hcp) rings capped by a spike complex, consisting of a trimer of valine–glycine repeat protein G (VgrG) proteins on which sits a conical proline–alanine–alanine–arginine repeat (PAAR) tip protein [5–7]. Effectors exist as either domains covalently linked to the lance constituents, called ‘evolved’ structural components, or cargo proteins that bind Hcp, VgrG or PAAR proteins in a non-covalent but specific manner [7].

**Fig. 1.**
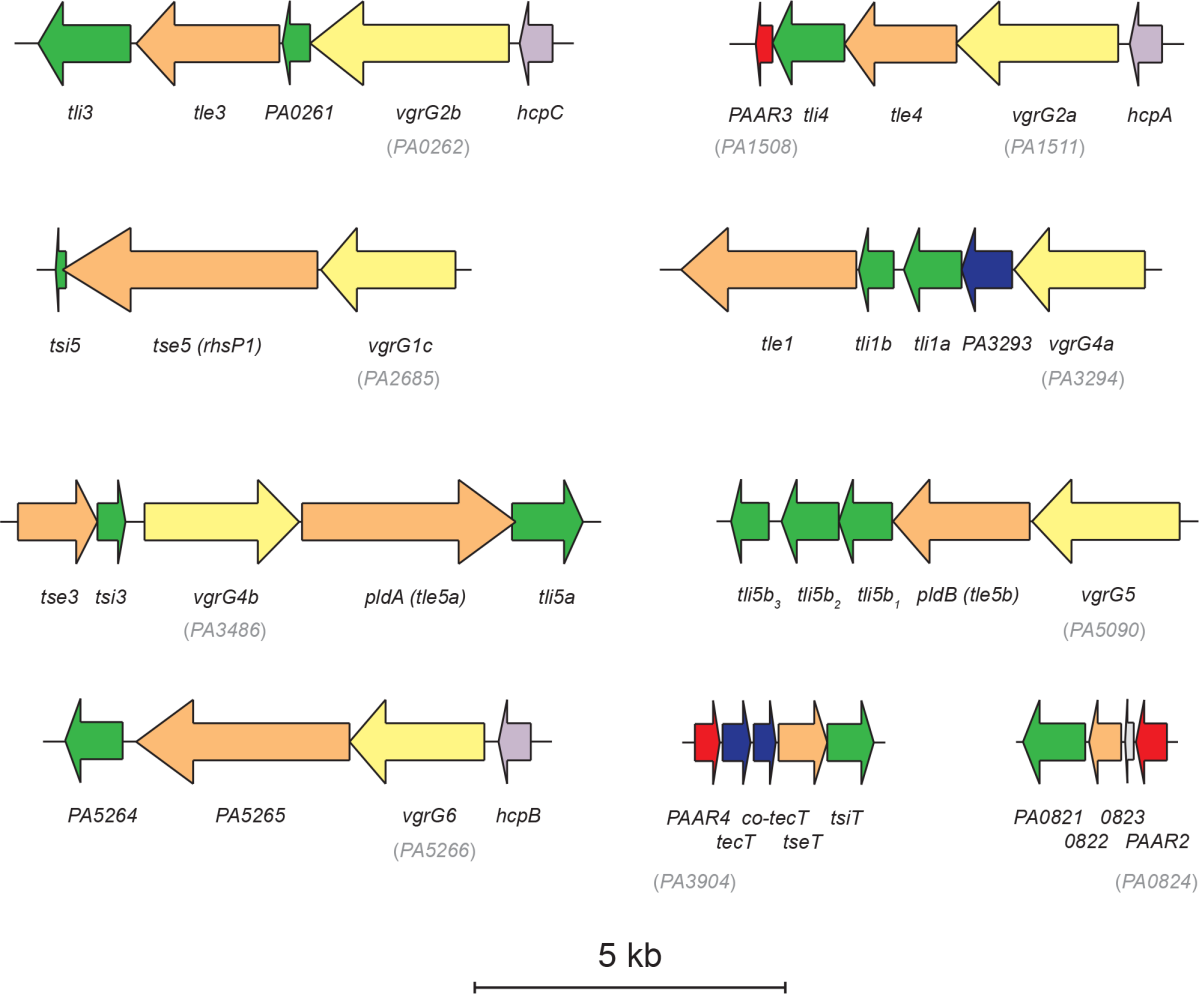
Schematic of the satellite *vgrG* and *PAAR* islands of *P. aeruginosa* PAO1. The *vgrG* and *PAAR* islands distal to the core T6SS gene clusters also encode charactersed or putative effector–immunity pairs. Genes encoding PAARs are in red, those encoding VgrG are in yellow, Hcp are in purple, effectors are in orange, immunity proteins are in green, chaperones are in dark blue and hypothetical proteins are in grey. The locus tag of each *vgrG* and *PAAR* gene is shown in parentheses. Scale bar shows 5 kb.

We have previously proposed an ‘à la carte’ delivery mechanism for the T6SS whereby effectors are recruited to the apparatus for secretion by specific spike components, a concept corroborated by others [8, 9]. Our understanding of the relationship between PAAR, VgrG and effector proteins has only recently developed through dissection of the effector repertoires of bacteria harbouring a single T6SS, namely *Vibrio cholerae *, *Serratia marcescens* and *Agrobacterium tumefaciens* [10–12]. *Pseudomonas aeruginosa* possesses three T6SSs, designated H1-, H2- and H3-T6SS, with antibacterial activity attributed to each [13–15]. Although the associations between evolved PAAR proteins and their cognate VgrGs are well established for the H1-T6SS, our knowledge of the make-up of the H2-T6SS spike complex is lacking [8, 9, 16]. No genes encoding spike proteins have been identified within the H2-T6SS locus in *P. aeruginosa* PAO1, although many satellite islands containing *vgrG* and *PAAR* genes are located distally on the chromosome (Fig. 1) [17]. Several VgrG proteins, including VgrG2a, VgrG2b, VgrG4b, and VgrG6, have been functionally associated with the H2-T6SS, with only VgrG4b having been characterised as being associated with a specific cargo effector, PldA [18–20]. Recently, Burkinshaw and co-workers determined that the PAAR4 tip protein is the cognate delivery device for the TseT nuclease effector of the H2-T6SS, which associates with VgrG4b or VgrG6 in the spike complex [19]. However, our understanding of the repertoire of VgrG–PAAR effector assemblies for the H2-T6SS is still in its infancy.

Here, we characterise a new cargo effector protein, Tle3, and identify the VgrG–PAAR subassembly responsible for its delivery by the H2-T6SS. Bioinformatic analysis of the *P. aeruginosa* genome also uncovers an unannotated open reading frame (ORF) encoding a PAAR protein bearing homology to the H2-T6SS-associated tip proteins. Use of bacterial competition assays and secretion assays dissects the capacity of the H2-T6SS to deliver multiple effector proteins and reveals a preferential delivery of the VgrG2b–PAAR3 spike subassembly. These data demonstrate that competition between spike complexes defines the effector payload of the H2-T6SS.

## Results

### Bioinformatics analysis of *P. aeruginosa* PAAR proteins

**Fig. 2.**
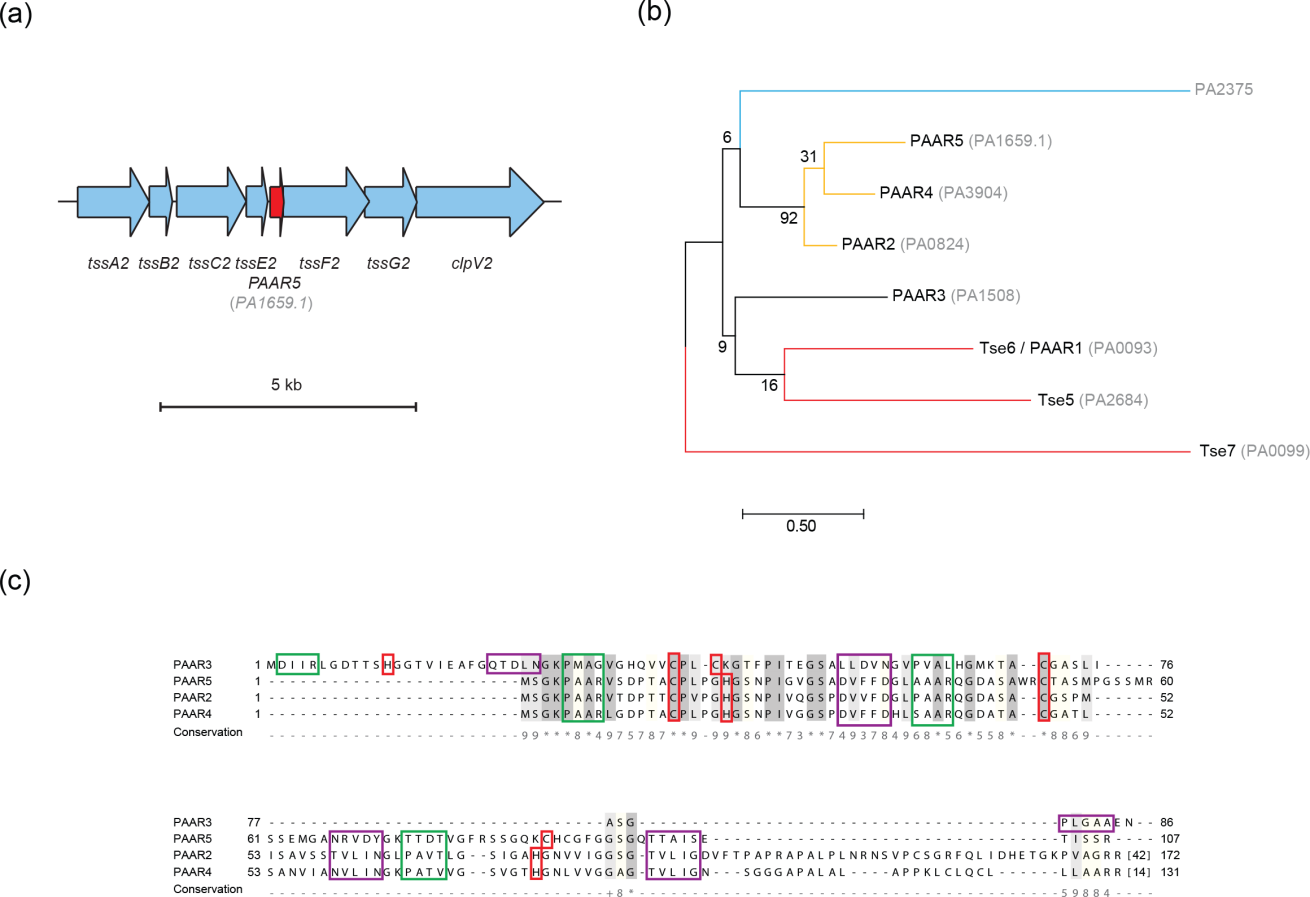
*In silico* analysis of PAAR proteins of *P. aeruginosa* PAO1. (a) Schematic of part of the *H2-T6SS* locus in *P. aeruginosa* PAO1. Genes encoding components of the H2-T6SS baseplate and sheath assemblies are shown in blue, with the newly identified *PA1659.1* ORF encoding the putative PAAR5 protein shown in red. Scale bar shows 5 kb. (b) Phylogenetic analysis of PAAR and PAAR-like domains in *P. aeruginosa* PAO1 using the maximum-likelihood method with 1000 bootstrap replicates. Bootstrap values are shown at the nodes. The associated locus tags of named PAAR-containing proteins are shown in grey. Tse7, PA2375 and Tse5 contain DUF4150, DUF4280 and cryptic PAAR-like domains, respectively, and thus do not have a numbered PAAR nomenclature. Proteins associated with a specific T6SS machinery, either empirically demonstrated or through genetic linkage, have their branches colour-coded: H1-T6SS-associated in red; H2-T6SS-associated in gold; and H3-T6SS-associated in blue. Scale bar represents the number of amino acid substitutions per site. (c)Multiple sequence alignment of the predicted H2-T6SS-associated PAAR proteins. PAAR protein sequences aligned using MAFFT. Light to dark grey shading indicates increasing conservation of residue identity as denoted by the conservation score below the alignment. PAAR motifs are highlighted with a green box, the predicted zinc-binding residues are encased in a red box and the residues modelled to interact with the cognate VgrG protein are bounded by a purple box. The number of omitted residues in gaps is shown in square brackets.

Effector delivery by the T6SS requires a functional spike complex, composed of a trimer of VgrG proteins capped by a PAAR protein [7, 21]. Seven proteins with PAAR or PAAR-like domains have been described in *P. aeruginosa* PAO1, four of which are canonical PAAR proteins (PAAR2–4 and PA2375) with no fused effector domains [7–9, 19]. Three effectors containing PAAR or PAAR-like domains (Tse5, Tse6 and Tse7) have been functionally associated with the H1-T6SS, while the *PA2375* gene, found in the H3-T6SS locus, encodes a member of the DUF4280 family, shown to be structural homologues of PAAR proteins [8, 9, 16, 22, 23]. *In silico* analysis of the *P. aeruginosa* PAO1 genome using a tblastn search with PAAR protein queries identified an unannotated ORF, designated with the locus tag *PA1659.1*, between the *tssE2* and *tssF2* genes within the *H2-T6SS* locus (Fig. 2a). The putative product of this gene is a polypeptide of 10.8 kD, comprising a single PAAR_CT_1 domain sharing 55 % sequence identity over 97 residues with the PAAR4 protein. Expanding this analysis to 89 complete *P. aeruginosa* genomes identified this ORF within the *H2-T6SS* locus in all strains except *P. aeruginosa* PA7 (Table S1, available in the online version of this article). We henceforth refer to this conserved putative tip protein as PAAR5, in line with the PAAR nomenclature recently put forth by Burkinshaw and colleagues [19].

Analysis of the phylogeny of all PAAR domains encoded in *P. aeruginosa* PAO1 revealed that PAAR5 clusters with PAAR4 and PAAR2 (Fig. 2b), both of which have recently been functionally associated with the H2-T6SS [19]. Since PAAR5 is encoded within the *H2-T6SS* locus, it is likely that the tip protein is a component of this specific T6SS machinery. The PAAR3 protein, encoded by *PA1508*, is encoded in the *vgrG2a* satellite island along with the Tle4–Tli4 effector immunity pair and an Hcp2 paralogue, HcpA. The genetic linkage of *PAAR3* with H2-T6SS-associated genes led us to hypothesise that PAAR3 is also functionally linked to the H2-T6SS (Fig. 1). Our laboratory amongst others has begun to identify the key residues at the PAAR–VgrG interface, determining the specificity of the PAAR–VgrG interaction [7, 8, 16, 19]. PAAR proteins display a conserved conical tertiary structure despite generally exhibiting low similarity at the amino acid level, yet alignment of PAAR2, PAAR3, PAAR4 and PAAR5 reveals high sequence identity (Fig. 2c). Since the residues of PAAR2, PAAR4 and PAAR5 that are predicted to lie at the VgrG interface are well conserved, this supports the notion that they form subassemblies with closely related VgrG proteins and furthermore are functionally associated with the H2-T6SS (Fig. 2c).

### PAAR3 is required for VgrG2b secretion

**Fig. 3.**
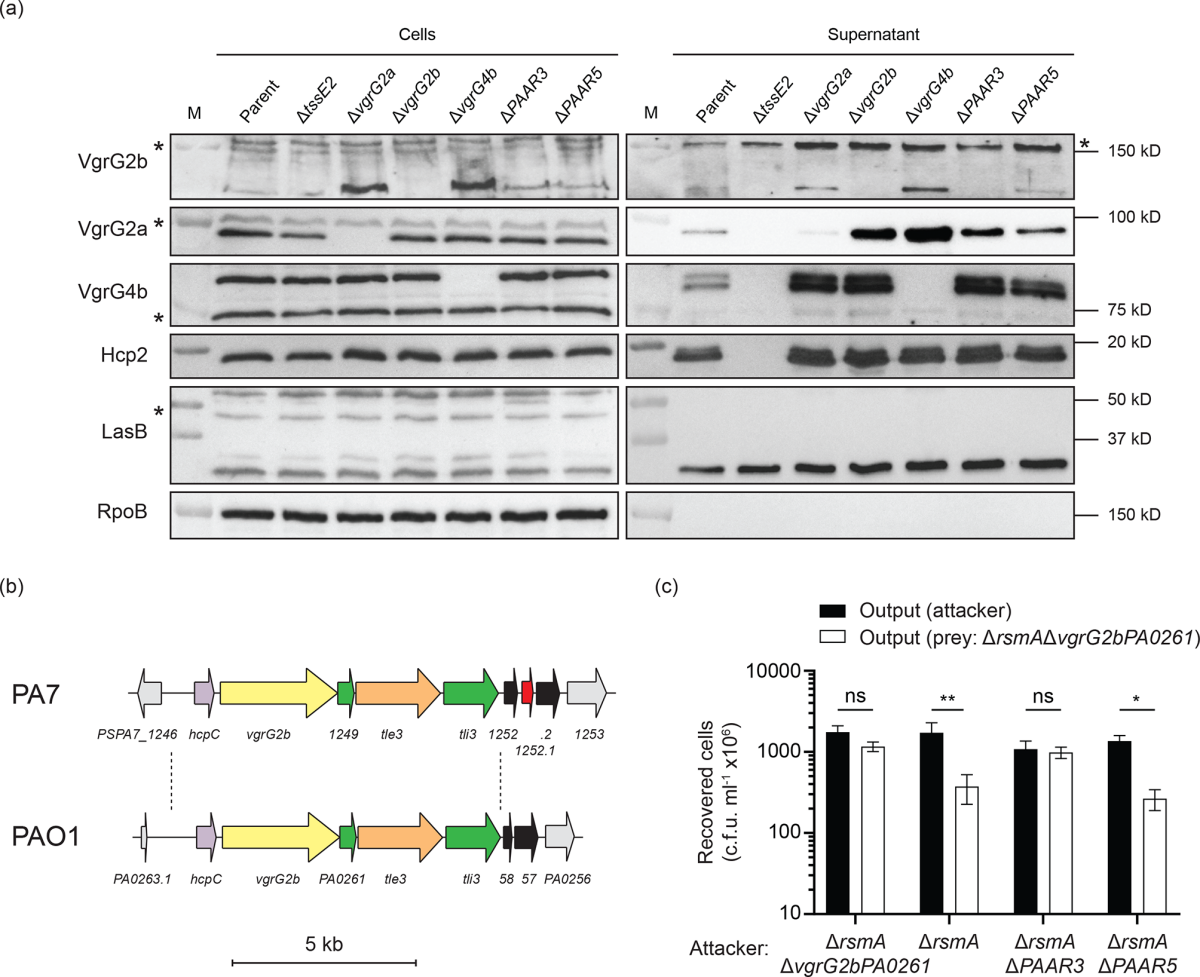
VgrG2b secretion requires the PAAR3 protein. (a) Immunoblot analysis of H2-T6SS structural components in the cellular and supernatant fractions of bacterial cultures. Polyclonal antibodies against Hcp2, VgrG2a, VgrG2b and VgrG4b were employed to assess the secretion of these proteins in PAO1Δ*rsmA* or derivative strains lacking spike components grown in H2-T6SS-conducive conditions. A double band was consistently detected by the anti-VgrG4b antibody in the supernatant fraction, possibly implying cleavage or modification of the secreted VgrG4b. Asterisks denote non-specific bands recognised by the polyclonal antibodies. RpoB and LasB immunoblots act as loading controls for the cellular and supernatant fractions, respectively. LasB is a type II secretion system effector, secreted independently of the T6SSs. RpoB also acts as a lysis control. Immunoblots are representative of three independent experiments. (b) Genomic context of the *vgrG2b* orthologue in *P. aeruginosa* PA7. The *vgrG2b* island, encompassing *hcpC* through *tli3*, is situated elsewhere on the chromosome relative to the locus in *P. aeruginosa* PAO1 and harbours a PAAR-encoding gene downstream, flanked by two putative transposase genes. Gene coloration is consistent with that in Fig. 1, with the addition of transposable elements in black. Scale bar corresponds to 5 kb. (c) Competitive growth outcome of the *P. aeruginosa* PAO1Δ*rsmA*Δ*vgrG2bPA0261* prey strain against attackers lacking the *PAAR3* or *PAAR5* genes. Competitive parity is represented by PAO1Δ*rsmA*Δ*vgrG2bPA0261* competing against itself, while the competition with the parental PAO1Δ*rsmA* strain acts as the positive control. The recovered output shows the mean of three independent experiments with the sem depicted with error bars. A two-way analysis of variance (ANOVA) with Sidak’s multiple comparisons test was used to determine statistically significant differences between the recovered prey and attacker strains for each competition assay (** *p* < 0.01; * *p* < 0.05; ns, not significant).

Amongst the four PAAR proteins that we propose to be associated the H2-T6SS, PAAR3 and PAAR5 are completely uncharacterised. To initiate their characterisation, we constructed strains lacking the *PAAR3* or *PAAR5* genes and probed the activity of the H2-T6SS in these genetic backgrounds. We have previously shown that Hcp2 and VgrG4b are secreted by the H2-T6SS of *P. aeruginosa* and thus monitored their secretion, and that of VgrG2a and VgrG2b, in the presence and absence of PAAR3 and PAAR5 [18]. In PAO1, HcpA, HcpB and HcpC are identical proteins encoded in the satellite islands of *vgrG2a*, *vgrG6* and *vgrG2b*, respectively (Fig. 1), and we refer to these tube proteins collectively as Hcp2, since the native proteins are indistinguishable. Secretion of Hcp2 is unaffected by the deletion of either *vgrG2a*, *vgrG2b*, *vgrG4b*, *PAAR3* or *PAAR5*, indicating that individually none of these spike and tip proteins is indispensable for H2-T6SS function (Fig. 3a). VgrG4b also appears to be secreted independently of this subset of VgrG and PAAR proteins, as none of *vgrG2a*, *vgrG2b*, *PAAR3* or *PAAR5* is essential for its secretion by the H2-T6SS (Fig. 3a). Likewise, VgrG2a is secreted independently of *vgrG2b*, *vgrG4b*, *PAAR3* or *PAAR5*, although its levels are slightly lowered in the absence of either PAAR protein. However, deletion of *PAAR3* does prevent secretion of VgrG2b, whereas this protein can still be detected in the supernatant of a Δ*PAAR5* strain (Fig. 3a).

Global analysis of complete *P. aeruginosa* genomes revealed that all strains encoding VgrG2b also contain the *vgrG2a* cluster in which PAAR3 is encoded, apart from *P. aeruginosa* PA7 (Table S1). This taxonomic outlier strain possesses few orthologues of VgrG proteins encoded by strain PAO1; however, the *vgrG2b* satellite island is present, albeit at a distinct chromosomal location (Fig. 3b) [24]. Since VgrG2b secretion absolutely requires PAAR3 in strain PAO1, we searched for a homologous PAAR protein within strain PA7 that would permit secretion of VgrG2b. A tblastn search revealed an ORF downstream of the *vgrG2b* cluster flanked by two putative transposase genes (Fig. 3b), which encodes a protein sharing 94 % sequence identity with PAAR3. We hypothesise that the insertion of this cassette compensates for the loss of the PAAR3-encoding *vgrG2a* satellite island from the genome of the PA7 strain by re-enabling secretion of VgrG2b. In all, these data suggest that PAAR3 is the cognate tip component for the VgrG2b spike protein.

We have recently discovered that the C-terminal domain of the evolved VgrG2b spike protein is a metallopeptidase-like antibacterial effector, whose detrimental activity is negated by the presence of its cognate immunity protein PA0261 (Wood *et al*., under revision). To verify that PAAR3 is required for the delivery of VgrG2b_C-ter_ into prey cells, we conducted a competition assay in H2-T6SS-conducive conditions between the prey strain lacking *vgrG2bPA0261* and attackers lacking either *PAAR3* or *PAAR5*. Whereas a Δ*PAAR5* attacker strain eliminates the prey strain at a similar efficiency to the parental attacker, deletion of *PAAR3* completely abolishes killing of the prey (Fig. 3d). This confirms that PAAR3 is required for VgrG2b delivery.

### Tle3 is an antibacterial effector delivered by the VgrG2b–PAAR3 spike complex

**Fig. 4.**
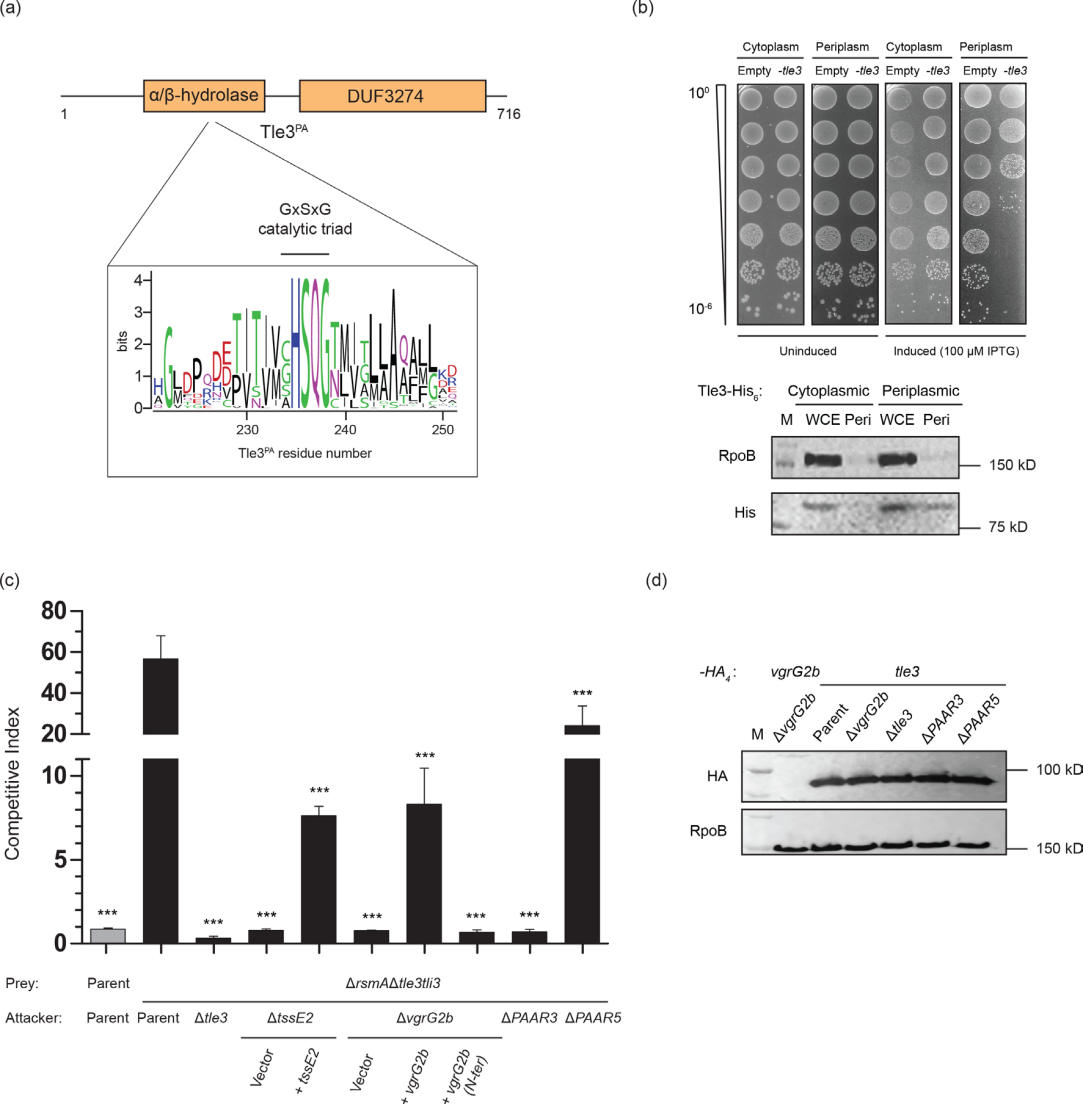
Tle3 is a periplasmic antibacterial toxin delivered by the VgrG2b-PAAR3 spike complex. (a) Domain schematic of the Tle3 protein from *P. aeruginosa* depicting the α/β-hydrolase and DUF3274 domains. A sequence logo of the predicted GXSXG consensus esterase motif of homologous proteins is shown below. (b) Heterologous production of Tle3 is toxic to *E. coli* when targeted to the periplasm. *Upper panel*: Serial dilutions of *E. coli* BL21 (λDE3) carrying pET28a-*tle3* or pET22b-*tle3* (for cytoplasmic or periplasmic effector production, respectively) or the empty vector equivalents were spotted on non-inducing (2 % glucose) and inducing (100 µM IPTG) media. The range of OD_600_ values for the inoculum is stated on the left. *Lower panel*: Immunoblot analysis of Tle3 production in the desired bacterial compartment. Whole cell extracts (WCE) and periplasmic fractions of *E. coli* BL21 (λDE3) pET28a-*tle3* and pET22b-*tle3* after growth in inducing conditions were probed using anti-His antibodies to detect the hexahistidine-tagged Tle3 protein. Antibodies against the RNA polymerase β-subunit RpoB were utilised to confirm no cytoplasmic contamination of the periplasmic fraction. Images are representative of three independent experiments. (c) Intraspecies competitive growth assay between a *P. aeruginosa* prey strain lacking the *tle3-tli3* cassette and various attacker strains. The competition index indicates the change in the attacker/prey strain ratio at the end of the assay relative to the input, where a value >1 represents a growth advantage to the attacker strain. Competition between the parental strain PAO1Δ*rsmA* and itself, shown in grey, acts as the internal control for competitive parity. Values denote the mean of three independent experiments, with error bars displaying the sem. Statistical significance of growth outcomes was determined by a one-way ANOVA followed by Dunnett’s multiple comparisons test, using the competition between the Δ*rsmA*Δ*tle3tli3* prey strain and the Δ*rsmA* parental strain as the comparator (*** *p* < 0.001). (d) Immunoblot showing the production of Tle3 in *P. aeruginosa* strains. The VgrG2b-HA_4_ construct acts as an antibody control, while RpoB is a loading control. Images are representative of three independent experiments.

The T6SS delivers a vast repertoire of antibacterial toxins, including nucleases, NAD(P)^+^ hydrolases, pore-forming effectors, peptidoglycan hydrolases and phospholipases [12, 25, 26] [15, 27]. Phospholipase toxins form the type VI lipase effector (Tle) superfamily, composed of Tle1-5 [15]. The characterised T6SS-associated phospholipases of *P. aeruginosa* PAO1 are Tle1, Tle4, PldA (Tle5a) and PldB (Tle5b), all of which display antibacterial activity on membrane phospholipids within the periplasm [14, 28] [15, 29], . Our laboratory amongst others has found that effector proteins are often encoded in the same locus as the VgrG proteins responsible for their delivery [8, 9, 16, 18, 30–33]. The product of the *PA0260* gene, encoded within the satellite island of the T6SS spike protein VgrG2b, is predicted to be a member of the uncharacterised Tle3 family with the consensus GXSXG catalytic motif within the α/β-hydrolase domain and a C-terminal DUF3274 domain of unknown function (Fig. 4a) [15]. Type VI effector proteins that act within the periplasmic compartment lack a native signal sequence to avoid self-intoxication, and heterologous production of Tle3 in *Escherichia coli* does not elicit toxicity (Fig. 4b). However, when artificially targeted to the periplasm by an N-terminal Sec-dependent signal peptide, Tle3 caused a 1000-fold decrease in cell viability.

Antibacterial toxins are invariably encoded adjacent to immunity proteins that neutralise the effector in donor and sister cells to prevent kin elimination. While upstream of *tle3* lies *PA0261*, encoding the VgrG2b_C-ter_ immunity protein (Wood *et al*. under revision), the gene downstream (*PA0259*) codes for a protein of unknown function, henceforth designated Tli3 (see below), the putative immunity protein to Tle3 (Fig. 1). Since Tle3 is encoded within the *vgrG2b* satellite island, we reasoned that this cargo effector may be delivered by the H2-T6SS, requiring VgrG2b as its cognate spike protein. We constructed a *P. aeruginosa* strain lacking the *tle3–tli3* cassette and assayed its contact-dependent competitive fitness with the parental strain under conditions in which the H2-T6SS is active. Indeed, the prey strain was robustly eliminated by the parent, whereas deletion of the *tle3* gene from the attacker abolished its ability to kill the prey strain (Fig. 4c). Delivery of the Tle3 toxin required both the spike protein VgrG2b and a functional H2-T6SS, as deletion of *vgrG2b* or the baseplate component gene *tssE2* rescued the survival of the prey strain. Complementation of these genes *in trans* partially restored killing by the attacker. We have previously defined the modular architecture of VgrG2b as consisting of the spike region (containing a gp27-like, gp5-like and DUF2345 domain), a transthyretin (TTR)-like fold and a C-terminal metallopeptidase effector domain (Wood *et al*., under revision). Expression of a construct encoding the N-terminal spike region of VgrG2b (gp27-gp5-DUF2345) in the Δ*vgrG2b* strain was unable to restore any prey killing, unlike the full-length *vgrG2b* gene, indicating that the C-terminal region (TTR-effector) of this evolved spike protein is necessary for Tle3 delivery (Fig. 4c). The TTR domain of VgrG1 from enteroaggregative *E. coli* is involved in the delivery of its cognate phospholipase effector, Tle1, so we predict that the TTR domain of VgrG2b is likely responsible for Tle3 delivery [31]. In accordance with our data implicating PAAR3 in the secretion of VgrG2b, we find that Δ*tle3tli3* is no longer susceptible to elimination by an attacker lacking this PAAR protein, whereas a Δ*PAAR5* attacker maintains a 25-fold competitive advantage (Fig. 4c). We therefore conclude that PAAR3 is required for the delivery of Tle3 by the H2-T6SS spike protein VgrG2b.

### Tli3 is the cognate periplasmic immunity protein to Tle3

**Fig. 5.**
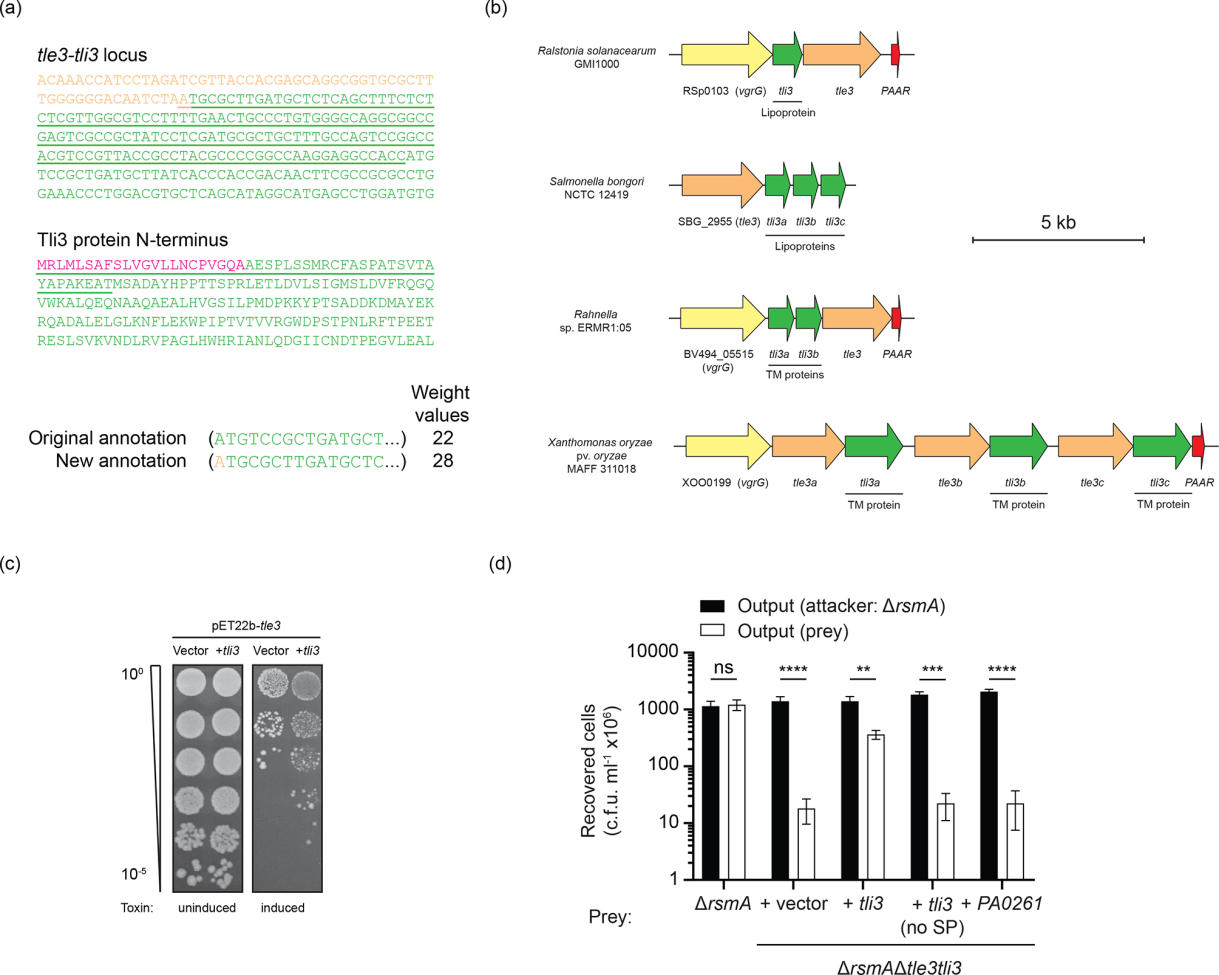
Immunity to Tle3 is conferred by the exported Tli3 family of proteins. (a) Analysis of the *tli3* gene reveals a putative upstream translational start site to produce a protein with a predicted N-terminal signal peptide. Upper panel: reannotation of the *tli3* gene (green) to the final nucleotide of the *tle3* (orange) ORF. The underlined sequence depicts the reannotation upstream of the previously suggested ATG start codon. Lower panel: primary sequence of the predicted Tli3 product from the reannotated gene. The underlined residues indicate the additional amino acids at the N-terminus, with the sequence in pink showing the predicted type I signal peptide as determined by SignalP 4.1. Below the alignments are the weight values of the nucleotides surrounding the putative ATG start codon in question according to the Kolaskar and Reddy method, where a score of 26 or higher indicates that the ATG is likely an initiator codon [34]. (b) *In silico* analysis of *tli3* homologues, showing the genetic architecture of diverse *tle3-tli3* loci. The nature of Tli3 export predicted by sequence analysis is annotated below the *tli3* gene to state whether it is a putative lipoprotein or transmembrane protein. Gene coloration is consistent with . (c) *E. coli* toxicity assay demonstrating the effect of Tli3 on Tle3-mediated toxicity. Serial dilutions of *E. coli* strains producing periplasmic Tle3 as well as *tli3* or its empty vector control are plated on media that repress (2 % glucose) or induce (100 µM IPTG) expression of the *tle3* construct. Images are representative of three independent experiments. (d) Outcome of a growth competition assay between *P. aeruginosa* PAO1Δ*rsmA* and its isogenic Δ*tle3tli3* derivative strain harbouring the empty vector or immunity gene constructs *in trans*. Competition between PAO1Δ*rsmA* and itself serves as the internal control for competitive parity. The recovered output displays the mean of three independent experiments, where error bars show the SEM. A two-way ANOVA followed by Sidak’s multiple comparisons test was used to determine the statistical significance of the difference in outcomes between competition of the parental strain with prey strains expressing *tli3* *in trans* or the empty vector (****P< 0.0001; ***P< 0.001; **P< 0.01; ns: not significant).

The cognate immunity proteins to phospholipase effectors harbour signal sequences to target them to the periplasmic space; however, the annotated *tli3* gene downstream of *tle3* is predicted to encode a cytoplasmic protein. However, closer examination indicates that expression of *tli3* from an alternative start site 150 bp upstream is predicted to produce a protein harbouring an N-terminal signal peptide (Fig. 5a). Comparison of the two putative start sites using the Kolaskar and Reddy algorithm [34] predicts that the the reannotated upstream start site is more likely to initiate translation (Fig. 5a). This apparent misannotation is found in all 79 instances of the *tli3* gene being identified in fully sequenced *P. aeruginosa* strains. Bioinformatics analyses of *tle3–tli3* loci in other proteobacteria show that the effector–immunity gene juxtaposition is maintained independently of the VgrG2b_C-ter_-PA0261 effector–immunity pair locus, reinforcing the notion that Tle3 is a classical cargo effector (Fig. 5b). Tli3 proteins could be identified as they bear a DUF2875 domain, which in some instances (such as in *P. aeruginosa *) is duplicated. Detailed inspection of the orthologous Tli3 coding sequences reveals a myriad of export signatures, such as putative lipoprotein type II signal peptides and N-terminal transmembrane helices, bolstering the predicted periplasmic localisation of the Tli3 protein family (Fig. 5b).

Heterologous expression of a construct encoding Tle3 artificially targeted to the periplasm along with Tli3 in *E. coli* shows that Tli3 improves the viability of bacteria producing the Tle3 effector, thus indicating that Tli3 is the cognate immunity protein of Tle3 (Fig. 5c). Moreover, while a *P. aeruginosa* prey strain lacking the *tle3–tli3* locus is robustly eliminated by the parental strain when placed in competition on solid media, provision of the full-length *tli3* gene *in trans* restores growth of the prey (Fig. 5d). If the sequence encoding the predicted signal peptide is omitted from the *tli3* coding sequence, prey elimination remains at the level of that of the prey strain harbouring either the empty vector or the unrelated *PA0261* immunity gene, demonstrating the requirement of the signal peptide for neutralisation of Tle3 by Tli3. In all, these results show that Tle3–Tli3 are an antibacterial phospholipase family effector–immunity pair acting in the periplasmic space.

### PAAR3 restricts the delivery of other H2-T6SS-dependent effectors

**Fig. 6.**
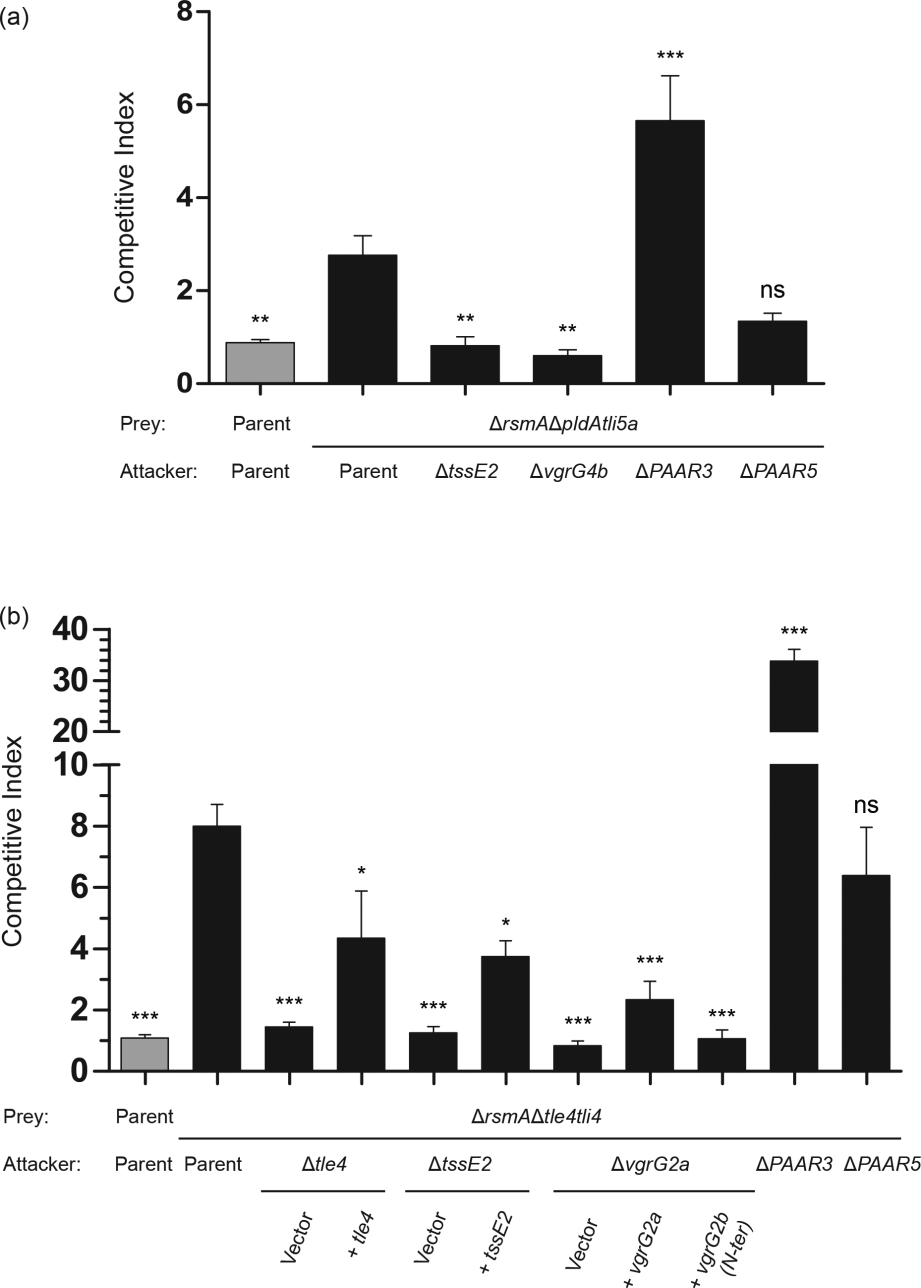
The PAAR3 spike restricts the delivery of other H2-T6SS-dependent hydrolytic effectors. Competitive growth assay between (a) PAO1Δ*rsmA*Δ*pldAtli5a* or (b) PAO1Δ*rsmA*Δ*tle4tli4* and various attacker strains. Competitive index values represent the mean of at least three independent experiments, with the sem shown by error bars. A competition assay between the PAO1Δ*rsmA* parental strain and itself acts as the internal control for competitive parity. Competitive growth outcomes that were statistically significant from the comparator competition between the parental strain and the prey strain lacking the effector–immunity pair of interest were determined using a one-way ANOVA with Dunnett’s multiple comparisons test and are denoted with asterisks (*** *p* < 0.001; ** *p* < 0.01; * *p* < 0.05; ns, not significant).

Several H2-T6SS-dependent effectors besides Tle3 have been characterised, including PldA and Tle4 [14, 15]. Here, we assessed the role of PAAR3 and PAAR5 in the delivery of the phospholipase effectors PldA and Tle4 through intra-species competition assays. Elimination of the PldA-susceptible prey Δ*pldAtli5a* is abolished when the attacker lacks its cognate VgrG4b delivery device or a functional H2-T6SS apparatus, as previously noted (Fig. 6a) [15, 18]. An attacker lacking *PAAR5* is still able to outcompete the prey strain to a similar extent as the parental strain; however, deletion of *PAAR3* greatly increases the ability of the attacker to eliminate the prey. This suggests that the presence of PAAR3 limits PldA delivery by the H2-T6SS.

Next, we demonstrated that Tle4-mediated elimination of a Δ*tle4tli4* prey strain in an H2-T6SS-dependent manner, first shown by Jiang and co-workers, could be achieved under the contact-dependent killing conditions developed in our laboratory (Fig. 6b) [29]. Due to the tendency of effector proteins such as PldA and Tle3 to utilise the VgrG encoded immediately upstream as their cognate delivery device, we hypothesised that VgrG2a, encoded adjacent to *tle4*, would perform such a role (Fig. 1). Indeed, deletion of *vgrG2a* abrogated the competitive advantage of the attacker, which could be partially restored through complementation of the full-length *vgrG2a* gene *in trans*. VgrG2a and VgrG2b display 99.5 % sequence identity across their gp27-gp5-DUF2345 spike regions, with only four residues differing between the N-terminal 757 residues of the two spike proteins. Unlike VgrG2b, VgrG2a is not considered to be an evolved VgrG and is predicted to harbour solely a C-terminal TTR domain, with just 25 % sequence identity to that of VgrG2b. Since the N-terminal spike region of VgrG2b is unable to complement the *vgrG2a* mutant despite its homology to the equivalent region of VgrG2a (Fig. 6b), we predict that the C-terminal TTR domain of VgrG2a is required for Tle4 delivery. Finally, in a similar manner to the delivery of the VgrG4b-dependent PldA toxin, we find that although deletion of *PAAR5* does not hamper Tle4 delivery, prey killing is once again enhanced by the absence of *PAAR3* (Fig. 6b). These data suggest that the delivery of H2-T6SS toxins by spike proteins other than VgrG2b, namely the cognate pairs VgrG4b–PldA and VgrG2a–Tle4, is restricted by the PAAR3–VgrG2b subassembly. This finding indicates that competition between PAAR–VgrG subassemblies of the H2-T6SS exists, and that at least under the conditions employed in this study, the VgrG2b–PAAR3 subassembly is able to outcompete the spike complexes formed by VgrG4b or VgrG2a. These spike complexes recruit cognate effectors to the T6SS baseplate to assemble a functional machine and therefore define the principal effectors secreted by a single apparatus.

## Discussion

In this study, we report the VgrG and PAAR dependence of a new antibacterial effector of the H2-T6SS of *P. aeruginosa* and begin to define the competition between spike subassemblies for this secretion system. The Tle3 toxin belongs to the phospholipase family of antibacterial effectors and its detrimental activity in the periplasm is neutralised by its cognate immunity protein Tli3, which is predicted to localise to this compartment. Tle3 requires the spike protein VgrG2b, encoded upstream, for its delivery and this in turn requires the PAAR3 tip protein. Intriguingly, our genetic approach reveals that the delivery of two other H2-T6SS-dependent phospholipase effectors, which utilise distinct cognate VgrG partners, is enhanced in the absence of PAAR3, therefore implying that the PAAR3-VgrG2b spike subassembly may outcompete other spike complexes for binding to the H2-T6SS apparatus.

Through a bioinformatics approach, we identify a conserved putative PAAR protein that is likely associated with the H2-T6SS based on both genetic linkage and homology to other PAAR proteins functionally linked to this system. Although this PAAR protein, which we designate PAAR5, does not appear to play a substantial role in the secretion of VgrG2a, VgrG2b or VgrG4b, several other spike proteins, namely VgrG4a, VgrG5 and VgrG6, are associated with the H2-T6SS. Recent work linked VgrG6 with PAAR4 [19]; however, the associated PAAR proteins for VgrG4a and VgrG5 remain elusive, and it is possible that PAAR5 assumes such a role. The high conservation of the predicted VgrG-binding interface of PAAR2, PAAR4 and PAAR5 may also permit functional redundancy, where non-cognate PAAR proteins may still deliver a particular spike protein with reduced efficiency.

We find that the delivery of Tle4 is VgrG2a-dependent, but that it does not require PAAR3, despite their genetic linkage. This is somewhat surprising due to their consistent synteny in *P. aeruginosa* strains, but is supported by the secretion of VgrG2a in the absence of *PAAR3* or *vgrG2b*, and VgrG2a-independent secretion of VgrG2b, which itself associates with PAAR3. Moreover, the authors of the seminal work characterizing PAAR proteins were unable to detect an interaction between PAAR3 and the VgrG2a spike in crystallographic analyses [7]. While we cannot fully discount that a VgrG2a–PAAR3 spike subassembly may exist, our findings strongly suggest that VgrG2a associates predominantly with a distinct tip protein.

PAAR proteins appear to be extremely important for T6SS functionality, as the PAAR–VgrG complexes form the puncturing device of the nanomachine [11]. The association of specific PAAR and VgrG proteins has previously been described in several bacteria, including *Serratia marcescens *, *Agrobacterium tumefaciens* and *P. aeruginosa *, while recent work has also highlighted the direct association of cargo effectors with PAAR proteins [8–11, 16, 19, 35]. In *S. marcescens *, three VgrG–PAAR subassemblies display differing delivery efficiencies, potentially due to relative expression levels or complex stability and prevalence [11]. The identity of the spike components appears to also influence the efficiency of the delivery of Hcp-dependent effectors; however, the precise reason for this remains elusive [9, 11].

Differential effector delivery has also been suggested within the H2-T6SS, where secretion of a PAAR4-dependent effector is enhanced in the absence of *vgrG2b* [19]. We further our understanding of this concept by showing that in the absence of PAAR3, which is required for VgrG2b and Tle3 secretion, the delivery of other VgrG–effector sets, namely VgrG2a–Tle4 and VgrG4b–PldA, appears to be elevated. These findings highlight that the competition for VgrG–PAAR recruitment to the T6SS apparatus defines the identity of the effector payload. Thus, the small conical PAAR ‘hat’ of the T6SS determines the identity of spike subassemblies, which recruit specific effector proteins for secretion. Comprehensive elucidation of the identities of the PAAR–VgrG–effector partnerships will enable determination of cargo delivery from amongst a broad arsenal, which could be harnessed for the selective bacterial delivery of therapeutic proteins into target cells.

## Methods

### Bacterial strains, growth conditions and plasmids

The strains and plasmids used in this study are listed in Table 1 and the oligonucleotides for cloning are provided in Table 2. Bacteria were cultured at 37 °C in lysogeny broth (LB) under agitation or on solid LB media unless stated otherwise. Media were supplemented with antibiotics and other compounds where appropriate at the following concentrations: kanamycin (50 µg ml^−1^), ampicillin (50 µg ml^−1^), streptomycin (50 µg ml^−1^), gentamicin (15 µg ml^−1^), isopropyl-β-d-thiogalactopyranoside (IPTG) (100 µg ml^−1^) for *E. coli *; carbenicillin (100 µg ml^−1^), streptomycin (1000 µg ml^−1^), gentamicin (50 µg ml^−1^) and 5-bromo-4-chloro-3-indolyl-d-galactopyranoside (X-gal) (100 µg ml^−1^) for *P. aeruginosa *. All deletion strains employed in this work are in-frame, markerless mutants and were constructed using the pKNG101 suicide vector as previously described [36]. The *E. coli* DH5α strain was used as the generic cloning host, while CC118λ*pir* and Sm10λ*pir* were used to mobilise the integrative plasmids pKNG101 and Mini-CTX into *P. aeruginosa* by three- and two-partner conjugation, respectively. *E. coli* BL21 (λDE3) was used for heterologous expression experiments.

**Table 1.** Strains and plasmids used in this study

Bacterial Strains	Description	Source
* P *. *aeruginosa* PAO1	Wild-type *P. aeruginosa* strain	Laboratory collection
* P. aeruginosa* PAO1Δ*rsmA*	Deletion of *PA0905*	[18]
* P. aeruginosa* PAO1Δ*rsmA*Δ*tssE2*	Deletion of *PA0905* and *PA1659*	This study
* P. aeruginosa* PAO1Δ*rsmA*Δ*vgrG2a*	Deletion of *PA0905* and *PA1511*	This study
* P. aeruginosa* PAO1Δ*rsmA*Δ*vgrG2b*	Deletion of *PA0905* and *PA0262*	This study
* P. aeruginosa* PAO1Δ*rsmA*Δ*vgrG4b*	Deletion of *PA0905* and *PA3486*	This study
* P. aeruginosa* PAO1Δ*rsmA*Δ*PAAR3*	Deletion of *PA0905* and *PA1508*	This study
* P. aeruginosa* PAO1Δ*rsmA*Δ*PAAR5*	Deletion of *PA0905* and *PA1659.1*	This study
* P. aeruginosa* PAO1Δ*rsmA*Δ*tle3*	Deletion of *PA0905* and *PA0260*	This study
* P. aeruginosa* PAO1Δ*rsmA*Δ*tle3tli3*	Deletion of *PA0905* and *PA0260-PA0259*	This study
* P. aeruginosa* PAO1Δ*rsmA*Δ*tle4*	Deletion of *PA0905* and *PA1510*	This study
* P. aeruginosa* PAO1Δ*rsmA*Δ*tle4tli4*	Deletion of *PA0905* and *PA1510-PA1509*	This study
* P. aeruginosa* PAO1Δ*rsmA*Δ*pldAtli5a*	Deletion of *PA0905* and *PA3487-PA3488*	This study
* E *. *coli* DH5α	F^–^ *endA1 glnV44 thi-1 recA1 relA1 gyrA96 deoR nupG purB20* φ80d*lacZ*ΔM15Δ(*lacZYA-argF*)U169, *hsdR17*(*r_K_* ^–^ *m_K_* ^+^), λ^–^	Laboratory collection
* E *. *coli* CC118λ*pir*	Δ(ara-leu) *araD* Δ*lacX74 galE galK-phoA20 thi-1 rpsE rpoB argE* (Ap^R^) *recA1 Rfr λpir*	[45]
* E *. *coli* Sm10λpir	*thi thr leu tonA lacY supE recA::RP4-2-Tc::Mu* (Km^R^) *λpir*	[46]
* E. coli* BL21 (λDE3)	F^–^ *ompT gal dcm lon hsdS_B_*(*r_B_^–^m_B_^–^*) λ(DE3 [*lacI lacUV5- T7p07 ind1 sam7 nin5*]) [*malB^+^*]_K-12_(λ^S^)	Laboratory collection
**Plasmids**		
Mini-CTX-*lacZ*	Integrative plasmid for inserting *lacZ* at a neutral site on the *P. aeruginosa* chromosome	[47]
pET28a	Expression vector, Km^R^	Novagen
pET28a-*tle3*	Expression plasmid producing Tle3 with a C-terminal hexahistidine tag, Km^R^	This study
pET22b	Expression vector with the PelB signal peptide to target proteins to the periplasm, Ap^R^	Novagen
pET22b-*tle3*	Expression vector producing Tle3 with a C-terminal hexahistidine tag, artificially targeted to the periplasm by an N-terminal signal peptide, Ap^R^	This study
pBBR1-MCS-4	Broad host range vector, Cb^R^	[48]
pBBR1-MCS-4-*tssE2*	Broad host range plasmid for constitutive expression of *tssE2*, Cb^R^	This study
pBBR1-MCS-4-*vgrG2b*	Broad host range plasmid for constitutive production of VgrG2b with a C-terminal quadruple HA tag, Cb^R^	This study
pBBR1-MCS-4-*vgrG2b*_N-ter_	Broad host range plasmid for constitutive production of the N-terminal canonical spike region of VgrG2b (1-757), Cb^R^	This study
pBBR1-MCS-4-*tle4*	Broad host range plasmid for constitutive production of Tle4 with a C-terminal quadruple HA tag, Cb^R^	This study
pBBR1-MCS-4-*vgrG2a*	Broad host range plasmid for constitutive production of VgrG2a with a C-terminal quadruple HA tag, Cb^R^	This study
pBBR1-MCS-4-*tle3*	Broad host range plasmid for constitutive production of Tle3 with a C-terminal quadruple HA tag, Cb^R^	This study
pBBR1-MCS-5	Broad host range vector, Gm^R^	[48]
pBBR1-MCS-5-*tli3*	Broad host range plasmid for constitutive production of Tli3 with a C-terminal HA tag, Gm^R^	This study
pBBR1-MCS-5-*tli3* (no SP)	Broad host range plasmid for constitutive production of Tli3 without the sequence coding for its signal peptide, with a C-terminal HA tag, Gm^R^	This study
pBBR1-MCS-5-*PA0261*	Broad host range plasmid for constitutive production of PA0261 with a C-terminal HA tag, Gm^R^	Wood *et al*., unpublished
pKNG101	Suicide vector, Sm^R^	[49]
pKNG101-(Δ*vgrG2a*)	Suicide plasmid to delete the *PA1511* locus, Sm^R^	This study
pKNG101-(Δ*vgrG2b*)	Suicide plasmid to delete the *PA0262* locus, Sm^R^	This study
pKNG101-(Δ*vgrG4b*)	Suicide plasmid to delete the *PA3486* locus, Sm^R^	[18]
pKNG101-(Δ*tle3*)	Suicide plasmid to delete the *PA0260* locus, Sm^R^	This study
pKNG101-(Δ*tle3tli3*)	Suicide plasmid to delete the *PA0260-PA0259* locus, Sm^R^	This study
pKNG101-(Δ*tle4*)	Suicide plasmid to delete the *PA1510* locus, Sm^R^	This study
pKNG101-(Δ*tle4tli4*)	Suicide plasmid to delete the *PA1510-PA1509* locus, Sm^R^	This study
pKNG101-(Δ*PAAR3*)	Suicide plasmid to delete the *PA1508* locus, Sm^R^	This study
pKNG101-(Δ*PAAR5*)	Suicide plasmid to delete the *PA1659.1* locus, Sm^R^	This study
pKNG101-(Δ*pldAtli5a*)	Suicide plasmid to delete the *PA3487-PA3488* locus, Sm^R^	This study

**Table 2.** Oligonucleotides used in this study

Primer Name	Purpose	Sequence
OAL3632	*tle3-His_6_*_F	AGAGGATCCGGCAAGGAGAGAAGCCATG
OAL3628	*tle3-His_6_*_R	GATGAATTCTAGTGGTGGTGGTGGTGGTGGATTGTCCCCCCAAAGCG
OAL1265	*vgrG2b*_F	AAAGGATCCATGCGTCAAAGGGACCTG
OAL2923	*vgrG2b*_N-ter__R	TACAAGCTTTCAGCCGACATG
OAL4192	*HA_4_*_F	CAAGAATTCAGCTAGCAGCGGAGTAGCGATGGTATATGC
OAL4098	*tle4-HA_4_*_F	ATAGGGCCCTCACAACAGCGACACGGAG
OAL4099	*tle4-HA_4_*_R	TCAGAATTCTTGCTAGCTGTCCCTCCTTTGTCATTAGGATG
OAL790	*tssE2-His_6_*_F	CGGAATTCCGCTGGTCGGCAAGCTGG
OAL3508	*tssE2-His_6_*_R	TTGGAGCTCTCAGTGGTGGTGGTGGTGGTGGCTGACCTTCACCTGGCC
OAL4095	*vgrG2a/b-HA_4_*_F	ATAGGGCCCGCCAGGAACAAGGAACGAT
OAL4096	*vgrG2b-HA_4_*_R	TCAACTAGTTGAATTCTTGTATCCCGTTGGGAAGTTTTTCAG
OAL4097	*vgrG2a-HA_4_*_R	TCAGCTAGCTGTGTTGGACTCCGTGTCG
OAL3333	*tli3-HA*_F	TTACTCGAGGGGGACAATCTAATGCGCTT
OAL3334	*tli3-HA*_R	GTTAAGCTTTTAGCACGCGTAGTCCGGCACGTCGTACGGGTATAAATCGTCCTGCCAGTCGG
OAL5193	*tli3*(no SP)*-HA_F*	ATGCTCGAGGAGAGAAGCCATGCGCTGCTTTGCCA
OAL2452	*tle3_*vector_F	TTTGCGCCGACATCATAACG
OAL5194	*tle3-HA_4__R*	GCTGCTAGCGATTGTCCCCCCAAAGCGC
OAL2575	pKNG101-(Δ*PAAR3*)Up_F	AAACAGCCTTCTTGAGCGAG
OAL2576	pKNG101-(Δ*PAAR3*)Up_R	TCAGTTTTCGATGTCCATCGCGGGTTGC
OAL2577	pKNG101-(Δ*PAAR3*)Dn_F	ATGGACATCGAAAACTGAAAAAGGGGGACGT
OAL2578	pKNG101-(Δ*PAAR3*)Dn_R	CTTTCCAGAGCTGCTTCCAC
OAL2579	pKNG101-(Δ*PAAR3*)Ext_F	AGAGGTTTGGAGTGGGAGTC
OAL2580	pKNG101-(Δ*PAAR3*)Ext_R	CGGGTATTTCTGGCCGAAC
OAL3366	pKNG101-(Δ*PAAR5*)Up_F	TGATCTAGACACAGATCATCGTCAGTGAC
OAL3367	pKNG101-(Δ*PAAR5*)Up_R	GTTTCACTGGGTTTGCCGGACATC
OAL3368	pKNG101-(Δ*PAAR5*)Dn_F	CGGCAAACCCAGTGAAACGATATCCAGCAGG
OAL3369	pKNG101-(Δ*PAAR5*)Ext_R	GTCTTCGGCGAACCCCAC
OAL3227	pKNG101-(Δ*pldAtli5a*)Up_F	GCGATCAAGATGCCGTTGAC
OAL3228	pKNG101-(Δ*pldAtli5a*)Up_R	TACTTCTTCCTTCTTCTGCAACATGGATCAGTC
OAL3229	pKNG101-(Δ*pldAtli5a*)Dn_F	CAGAAGAAGGAAGAAGTACTGCCCCGCC
OAL3230	pKNG101-(Δ*pldAtli5a*)Dn_R	TTCTTCACCAGCATCTCGGT
OAL3231	pKNG101-(Δ*pldAtli5a*)Ext_F	CCGGGCAGAAGATGGTGATC
OAL3232	pKNG101-(Δ*pldAtli5a*)Ext_R	AGGACGATGCAATTGGTGGT
OAL3663	pKNG101-(Δ*tle3*)Dn_F	AACGATAGCAGCCTCCTAGTCGGGACACAAACCATCC
OAL3664	pKNG101-(Δ*tle3*)Dn_R	TATACTAGTTCCTCCGGAGTGAAGCGT
OAL3629	pKNG101-(Δ*tle3*)ExtR	CAAGTCAGGGTTGCGTTCGA
OAL3289	pKNG101-(Δ*tle3* / Δ*tle3tli3*)Up_F	CATTCTAGACCAACGGGATACTGACCAATGAA
OAL3290	pKNG101-(Δ*tle3* / Δ*tle3tli3*)Up_R	GAGGCTGCTATCGTTCATGGCTTCTCTCCTTGC
OAL3291	pKNG101-(Δ*tle3tli3*)Dn_F	ATGAACGATAGCAGCCTCAAACCCAGC
OAL3292	pKNG101-(Δ*tle3tli3*)Dn_R	TGAACTAGTTTTCGTGTGCCTAGTCGTGG
OAL3293	pKNG101-(Δ*tle3* / Δ*tle3tli3*)Ext_F	CCGGGAAAGACGTTGAAGGA
OAL3294	pKNG101-(Δ*tle3tli3*)Ext_R	GTAGGTTCGGATGGCGGTAG
OAL2049	pKNG101-(Δ*tle4* / Δ*tle4tli4*)Up_F	GCCGGGCAGAAGGTGGTGATCAAC
OAL2050	pKNG101-(Δ*tle4*)Up_R	TCCTTTGTCGCTGCTCATGTGTTGGAC
OAL2051	pKNG101-(Δ*tle4*)Dn_F	ATGAGCAGCGACAAAGGAGGGACATGA
OAL2052	pKNG101-(Δ*tle4*)Dn_R	AGCCCTAGATATCTCCACAGCATGT
OAL2053	pKNG101-(Δ*tle4* / Δ*tle4tli4*)Ext_F	CCGCAGCAAACCCTCCAG
OAL2054	pKNG101-(Δ*tle4*)Ext_R	AATGTTTCCATACCTGCAAACGTG
OAL2587	pKNG101-(Δ*tle4tli4*)Up_R	CTATAGGTCGCTGCTCATGTGTTGGACTCCGTG
OAL2588	pKNG101-(Δ*tle4tli4*)Dn_F	ATGAGCAGCGACCTATAGGAGCAACCC
OAL2584	pKNG101-(Δ*tle4tli4*)Dn_R	ATCAGCCATGGGTCTTTGC
OAL2586	pKNG101-(Δ*tle4tli4*)Ext_R	GTTTTCAGCGACCCCTACCTC
OAL3360	pKNG101-(Δ*tssE2*)Up_F / (Δ*PAAR5*)Ext_F	TGATCTAGAATCGAGACCAAGATCCCCAC
OAL3361	pKNG101-(Δ*tssE2*)Up_R	ACCTTCACCCTGCCGTATCCAGTCATG
OAL3362	pKNG101-(Δ*tssE2*)Dn_F	ATACGGCAGGGTGAAGGTCAGCTAAGGA
OAL3363	pKNG101-(Δ*tssE2*)Dn_R	TCAACTAGTAACTCGTCGTCCAGCTTCTG
OAL3364	pKNG101-(Δ*tssE2*)Ext_F	CTTCGCCAAGTATCGCTGGT
OAL3365	pKNG101-(Δ*tssE2*)Ext_R / (Δ*PAAR5*)Dn_R	TCAACTAGTCAGCAGGCACAGGTAGAGC
OAL2631	pKNG101-(Δ*vgrG2a*)Up_F	TGATGGTCCAGGGCTTCAAC
OAL2632	pKNG101-(Δ*vgrG2a*)Up_R	CGCTGTTGTGTTGACGCATGGATCGTTC
OAL2633	pKNG101-(Δ*vgrG2a*)Dn_F	ATGCGTCAACACAACAGCGACACGGAG
OAL2137	pKNG101-(Δ*vgrG2a*)Dn_R	GCTGACGATGTTATCCAGTC
OAL2634	pKNG101-(Δ*vgrG2a*)Ext_F	ACTTTCCTTCACCCTGGGCC
OAL2590	pKNG101-(Δ*vgrG2a*)Ext_R	CACCAGCCCTCCCATCGCATG
OAL2486	pKNG101-(Δ*vgrG2b*)Up_F	TGACCTCCGGCGAGCG
OAL2487	pKNG101-(Δ*vgrG2b*)Up_R	TCAGTATCCTTGACGCATGGATCGTTCCTTG
OAL2488	pKNG101-(Δ*vgrG2b*)Dn_F	ATGCGTCAAGGATACTGACCAATGAAATGCAAG
OAL2489	pKNG101-(Δ*vgrG2b*)Dn_R	GTCGAGCAGCATCCAGTTGC
OAL2490	pKNG101-(Δ*vgrG2b*)Ext_F	CGGCAACACCTATCAGGAAG
OAL2491	pKNG101-(Δ*vgrG2b*)Ext_R	CTCTCCTTGCCGCTCCTC

F, forward primer; R, reverse primer. Restriction enzyme sites are underlined.

### Secretion assays and immunoblot analysis

Overnight cultures of *P. aeruginosa* cultures were diluted to OD_600_ 0.1 in 25 ml tryptic soy broth (TSB) and grown at 25 °C in TSB for 8 h with agitation. To recover secreted bacterial proteins, cultures were centrifuged at 4000 **﻿** ***g*** at 4 °C for 10 min three times successively, carrying forward the uppermost supernatant each time to eliminate cellular contamination. Proteins were precipitated with 10 % trichloroacetic acid supplemented with 0.03 % sodium deoxycholate overnight at 4 °C. The precipitate was pelleted by centrifugation at 16 000 ***g*** for 30 min at 4 °C before washing with cold 90 % acetone and centrifugation once more. The washed pellet was air-dried and resuspended in Laemmli buffer to an equivalent of OD_600_ of 20. Sodium dodecyl sulfate/polyacrylamide gel electrophoresis and immunoblotting was conducted as previously described [37]. Here, polyclonal rabbit anti-VgrG2a [20], anti-VgrG2b [18], anti-VgrG4b [18], anti-Hcp2 [38] and anti-LasB (gift from Romé Voulhoux) antibodies were used at a 1 : 1000 dilution, along with monoclonal mouse anti-RpoB (1 : 5000, Neoclone), anti-HA (1 : 1000, Biolegend) and anti-His_6_ (1 : 1000, Sigma) antibodies.

### Periplasmic fractionation

*E. coli* cultures producing Tle3 or its periplasmic-targeted fusion were grown to an OD_600_ of 0.6 before the induction of construct expression with 100 µM IPTG. Three hours post-induction, cells were harvested by centrifugation at 8000 ***g*** at 4 °C. Pellets were normalised to an OD_600_ of 20, resuspended in 200 µl spheroplast buffer (200 mM Tris-HCl pH 8.0, 500 µM EDTA and 500 mM sucrose) containing 50 µg hen egg-white lysozyme (Sigma) and incubated on ice for 15 min. Then, 720 µl spheroplast buffer was diluted 1 : 1 with distilled water and added to the spheroplasts. The spheroplasts were separated from the periplasmic fraction by centrifugation at 5000 ***g*** for 10 min at 4 °C and the isolated periplasmic fraction was centrifuged once more to remove residual spheroplasts. The periplasmic fraction was adjusted to the equivalent of an OD_600_ of 10 in Laemmli buffer.

### Bacterial toxicity and competition assays

*E. coli* cells producing Tle3 and Tli3 constructs were grown overnight and normalised to an OD_600_ of 1 before serial dilution in phosphate-buffered saline (PBS). Dilutions were spotted on solid media containing the appropriate antibiotics and inducer (100 µM IPTG) or repressor (2 % glucose) compounds before being air-dried and grown at 37 °C for 24 h. For *P. aeruginosa* intra-species competition assays, overnight cultures were normalised to an OD_600_ of 3 and washed with PBS. Attacker and prey strains were mixed at a 1 : 1 ratio, centrifuged at 8000 ***g*** for 3 min and resuspended in 100 µl PBS. Five microlitres of the competition mixtures was spotted on low salt LB agar (10 g bactopeptone, 5 g yeast extract, 30 g agar l^−1^), dried and incubated at 25 °C for 24 h. Spots were scraped up into 1 ml PBS and serially diluted for plating on LB agar containing 100 µg ml^−1^ X-gal for the discrimination of attacker and prey strains, since the latter harbour mini-CTX-*lacZ* on the chromosome.

### *In silico* analyses

Nucleotide sequences for the *P. aeruginosa* genome were retrieved from www.pseudomonas.com [39] and queried using blastn and tblastn analyses [40]. MAFFT [41] was used for all amino acid sequence alignments and the phylogeny of PAAR domains was analysed with mega7 [42] using the maximum-likelihood method of tree generation with 1000 bootstrap replicates. The sequence logo for the consensus catalytic motif of Tle3 homologues was generated using the WebLogo server [43] and SignalP 4.1 was used for the prediction of signal peptides [44]. All statistical analyses described in this work were conducted with Prism 8.0 software (GraphPad).

## Supplementary Data

Supplementary material 1
